# The common structure of mentalizing

**DOI:** 10.1371/journal.pone.0332722

**Published:** 2025-09-26

**Authors:** Ahmad Asgarizadeh, Reihaneh Shoumali, Maryam Tahan

**Affiliations:** 1 Faculty of Education and Psychology, Shahid Beheshti University, Tehran, Iran; 2 Hillan Research Center, Tehran, Iran; 3 Faculty of Psychology and Education, University of Tehran, Tehran, Iran; Universita degli Studi Europea di Roma, ITALY

## Abstract

The primary goal of this research was to investigate the common factor structure of mentalizing by combining items from pre-existing validated tools, cross-validating the resulting structure, and exploring its associations with relevant constructs. Three sequential studies were conducted using community-dwelling samples (total *N* = 947). Study 1 used exploratory factor analysis on a merged item pool derived from eight measures of mentalizing. Study 2 utilized exploratory structural equation modeling to replicate the extracted structure and investigated its association with psychological dysfunction. Study 3 performed cross-validation of the factor structure and provided criterion-related validity by examining relations with markers of psychopathology and well-being. Factor analyses provided a 10-factor solution that covered distinct facets of mentalizing. Some factors, especially Nonmentalizing-Self and Emotion/Impulse Dysregulation, were strong predictors of dysfunction and psychopathology. Notably, after controlling for positive self-evaluation, individuals reporting greater confidence in understanding others’ minds (Mindreading Self-Concept) showed poorer psychological functioning (*β* = 0.157, *p* = .001), in line with theoretical emphasis on humility as a component of genuine mentalizing. The resulting 10-factor structure provides a framework to potentially differentiate between adaptive and maladaptive mentalizing, distinguish its components along the self–other continuum, and discriminate authentic mentalizing processes from subjective assessments of one’s mentalizing capacity.

## Introduction

Understanding and making sense of one’s own and others’ mental states is essential to adaptive personal and social functioning [1]. Such capacity, termed mentalizing or reflective functioning, is a complex construct characterized by four primary dimensions, each corresponding to different neural processes [2]: (1) the capacity to reflect on one’s own mental states versus those of others (self–other), (2) a deliberate, slow process requiring verbal reflection, awareness, and effort versus rapid, reflexive processes with minimal conscious attention (controlled–automatic), (3) reliance on external behavior versus internal experience as the source of inferences (external–internal), and (4) the ability to name and reason about mental states versus the capacity to understand and resonate with the emotional qualities of those states (cognitive vs. affective). Such imbalances, often triggered by heightened emotional arousal, can cause individuals to regress to more primitive, prementalizing modes of psychological functioning characteristic of earlier developmental phases before the full consolidation of mentalizing abilities [3]. Effective mentalizing could promote mental health [4], whereas mentalizing deficits are associated with various psychological difficulties [5,6].

A range of measurement methodologies, such as interviews, self-report instruments, and experimental tasks, have been created to assess mentalizing [7]. Nevertheless, each methodology has inherent limitations in fully capturing the complexity of this construct. For instance, the gold standard of interview-based measures, the Reflective Functioning Scale [8], still lacks evidence on some measurement properties [9]. In addition, it demands extensive training and a time-consuming administration/scoring process, which restricts its feasibility for clinical as well as research purposes. Task-based measures also suffer from notable limitations affecting their validity. A major problem is that ceiling effects can often be found, particularly in non-clinical populations, limiting the ability to differentiate levels of mentalizing sophistication [10,11]. Additionally, these measures tend to emphasize cognitive/other-oriented aspects of mentalizing while neglecting affective/self-oriented components that are essential in clinical contexts. Perhaps most importantly, the strong correlation between task performance and general cognitive ability calls their discriminant validity into question, suggesting these measures may primarily assess cognitive abilities rather than mentalizing specifically [12].

Self-report tools are more suitable for large-scale studies, as they provide practical advantages, namely cost-effectiveness and ease of administration. While several self-report measures of mentalizing have been developed, issues related to their reliability, validity, and comprehensiveness remain unresolved [13]. Among the most widely employed instruments are the Mentalizing Questionnaire [MZQ; 14], the Reflective Functioning Questionnaire [RFQ; 15], and the Mentalization Scale [MentS; [16]. The 15-item MZQ addresses the affective dimension well but disproportionately emphasizes self-related mentalizing with insufficient assessment of other-oriented processes. In fact, a direct content analysis shows that 12 of the items pertain to mentalizing the self, while only 3 assess mentalizing others. Additionally, the MZQ has frequently shown strong correlations (*r* ≈ .60) with emotion dysregulation measures [e.g., 17], questioning its discriminant validity.

The 8-item RFQ, designed to screen mentalizing through certainty and uncertainty about mental states, has been critiqued for its conceptual validity, psychometric structure, and scoring system [18,19]. It primarily targets self-related reflections while inadequately capturing other-related mentalizing, disproportionately focuses on uncertainty (7/8 items), and may not accurately measure the maladaptive aspect of certainty contrary to its developers’ intentions [18]. Lastly, The 28-item MentS measures three dimensions (self-related and other-related mentalizing, and motivation to mentalize), offering a more balanced self–other assessment than the RFQ and MZQ. Nevertheless, the MentS still shows certain limitations: it does not assess mentalizing under stress conditions when this capacity typically deteriorates, it includes items about general interest in psychology and values that may not directly reflect mentalizing capacity (e.g., items number 17 and 28), and most critically, its other-related dimension (aimed to capture healthy mentalizing) is positively associated with narcissistic features [20], suggesting issues with conceptualization and/or item formulation.

While we have focused our criticisms on these three widely used measures, it is important to note that several other self-report instruments developed more recently (e.g., Four-item Mentalising Questionnaire and Mentalizing Emotions Questionnaire) are also characterized, to varying degrees, by similar conceptual challenges and/or incomplete operationalization of the construct [13]. Collectively, these measures do not fully represent the contemporary definitions of mentalizing adequately. None fully addresses all four dimensions, and most provide inadequate coverage of normal vs. pathological mentalizing. The development of a more theoretically comprehensive framework that can better assess the multidimensional nature of mentalizing across its normal and pathological manifestations is thus paramount. Furthermore, it is important to acknowledge that the existing item pool largely neglects certain theoretically important aspects of mentalizing (such as body-related mentalizing).

The present paper describes three studies conducted to investigate the common factor structure of mentalizing using an integrated item pool from existing measures. Following a sequential approach, we first explored the factor structure of an integrated item pool created from existing measures of mentalizing (Study 1), then confirmed this structure and examined its relationship with dysfunction (Study 2), and finally cross-validated the structure while establishing associations with relevant clinical constructs (Study 3). We aimed to address two key questions: (1) What is the empirical factor structure of mentalizing when assessed more comprehensively, and (2) How do these empirically derived components relate to maladaptive functioning and psychopathology?

## Study 1

### Objectives

The primary objective of this study was to evaluate the potential factor structure emerging from an integrated item pool of existing self-report mentalizing measures. This process involved identifying relevant measures, ensuring cultural adaptation and translation accuracy, and conducting exploratory factor analysis (EFA).

## Materials and methods

### Procedures, participants, and measures

To identify relevant measures, On August 10^th^, 2024, we conducted a systematic search of SCOPUS, Web of Science, and PubMed using keywords related to mentalizing and assessment (“mentalizing,” “mentalization,” “reflective function,” “instrument,” “scale,” “questionnaire,” “inventory,” “validity,” “reliability,” “psychometric”). Published articles were included if they were written in English and described the original versions of self-report measures (i.e., abbreviated forms and cultural adaptations were excluded). The initial search identified 11 potentially relevant measures. However, to ensure the conceptual coherence of the item pool prior to any statistical analysis, we conducted an a priori review of all item contents. Based on this qualitative review, three instruments were excluded: the Mentalized Affectivity Scale (which captures a construct subtly distinct from general mentalizing), the Mentalizing Values Scale (which focuses on the specific valuation of mentalizing as a virtue), and the Failure to Mentalize Trauma Questionnaire (which focuses on mentalizing in the narrow context of traumatic experiences). The eight included measures are overviewed in Table 1.

**Table 1:** Brief descriptions of the included mentalizing measures.

Measure	# Items	Subscales
Mentalization Questionnaire [MZQ; 14]	15	Two structures are suggested: - Single-factor solution - Four-factor solution: (1) Refusing self-reflection, (2) Emotional awareness, (3) Psychic equivalence mode, and (4) Regulation of affect
Reflective Functioning Questionnaire [RFQ; 15]	8	Two-factor solution: (1) Certainty about mental states and (2) Uncertainty about mental states
Mentalization Scale [MentS; 16]	28	Three-factor solution: (1) Self-related mentalization, (2) Other-related mentalization, and (3) Motivation to mentalize
Certainty About Mental States Questionnaire [CAMSQ; 21]	20	Two-factor solution: (1) Self-certainty and (2) Other-certainty
Multidimensional Mentalizing Questionnaire [MMQ; 22]	33	Six-factor solution: (1) Reflexivity, (2) Ego-strength, (3) Relational attunement, (4) Relational discomfort, (5) Distrust, and (6) Emotional dyscontrol.
Four-Item Mentalising Index [FIMI; 23]	4	Single factor solution
Interactive Mentalizing Questionnaire [IMQ; 24]	24	Three-factor solution: (1) Self-self, (2) Self-other, and (3) Other-self
Mentalizing Emotions Questionnaire [MEQ; 25]	16	Three-factor solution: (1) Self, (2) Communicating, and (3) Other,

The eight retained measures were compiled into a single battery. Apart from the RFQ, MZQ, and MentS, which were previously translated into Persian [26,27], rest of the item pool was independently translated into Persian by two of the authors. All authors inspected and collaboratively resolved the translation discrepancies. Next, two native speakers back-translated the items into English. Back-translated items were compared against the original items, and incongruencies were addressed. For the combined measure, we selected a four-point scale (1 = *Mostly untrue*, 2 = *Somewhat untrue*, 3 = *Somewhat true*, 4 = *Mostly true*). The finalized item pool and sociodemographic questions were uploaded to an online survey platform (Porsline.com), and the respective URL was distributed via popular social media platforms in Iran, including Telegram, WhatsApp, Instagram, and Twitter. A sociodemographic survey was also administered to collect participants’ information regarding sex, age, relationship status (single, partnered, or married), educational attainment (from middle school through doctoral/medical degrees), university enrollment, employment situation (working or not working), and mental health status (whether they were diagnosed and receiving psychiatric/psychological treatment, or had no diagnosis). Participant data were collected from November 15^th^, 2024 to December 12^th^, 2024.

To address invalid responses common in online administration [28], quality screening was conducted using instructed-item responses (≥ 1 careless response), response time (< 2 seconds per item), maximum longstring (identical answers to more than half of the items), and person-total correlations (negative values) [29,30]. From 215 initial responses, 25 were flagged as careless, resulting in a final sample of 190 community-dwelling adult participants (M_age_ = 34.82, SD_age_ = 12.22, Range = 18–70; see Table 2 for the sociodemographic characteristics of the samples). No missing data were present.

**Table 2:** Sociodemographic characteristics of participants.

Characteristics		Study 1 (*N* = 190)		Study 2 (*N* = 451)		Study 3 (*N* = 306)	
		Frequency	Percentage	Frequency	Percentage	Frequency	Percentage
Sex	Female	133	70	360	79.8	248	81
	Male	57	30	91	20.2	58	19
Age	18-24	24	12.6	50	11.1	106	34.6
	25-34	99	52.1	99	22	56	18.3
	35-44	31	16.3	146	32.4	67	21.9
	45-54	15	7.9	101	22.4	52	17
	55<	21	11.1	55	12.2	25	8.2
Relationship status	Single	78	41.1	147	32.6	130	42.5
	In a relationship	27	14.2	25	5.5	37	12.1
	Married	79	41.6	253	56.1	127	41.5
	Widowed	6	3.2	26	5.8	12	3.9
Level of education	Middle school	4	2.1	12	2.7	16	5.2
	High school	21	11.1	94	20.8	95	31
	Associate	9	4.7	29	6.4	28	9.2
	BSc	73	38.4	168	37.3	103	33.7
	MSc	65	34.2	117	25.9	51	16.7
	PhD/MD	18	9.5	31	6.9	13	4.2
University enrollment	Students	45	23.7	62	13.7	71	23.2
	Non-students	145	76.3	389	86.3	235	76.8
Employment status	Employed	133	70	224	49.7	143	46.7
	Unemployed	57	30	227	50.3	163	53.3
Diagnostic Status	Diagnosed	21	11.1	46	10.2	24	7.8
	Non-diagnosed	169	88.9	405	89.8	282	92.2

All studies were conducted in accordance with the Declaration of Helsinki and participants provided digital, written informed consent prior to participation. The research protocol was reviewed and approved by the Ethics Committee of the Raftar Cognitive Neuroscience Research Center at Shahid Beheshti University, which granted an exemption from full ethical review on March 17, 2024. This approval confirmed the study’s minimal risk design, involving an anonymous online self-report survey with adult participants, and the acceptance of the informed consent procedures and data protection measures.

## Analysis strategy

All analyses were conducted using IBM SPSS [v. 27; 31] and packages psych [v. 2.4.12; 32] and EGAnet [v. 2.0.7; 33] in RStudio [v. 2024.09.0; 34]. Item-level statistics were examined, favoring item retention to preserve the measure’s content coverage, and items with excessive non-normality [absolute skewness/kurtosis > 2; 35], negative item-total correlations, or high redundancy (polychoric *r* > .70) were identified for potential removal.

Factor structure was investigated using exploratory factor analysis with polychoric correlations. After confirming data factorability through the Kaiser-Meyer-Olkin measure and Bartlett’s test of sphericity, we determined the optimal factor number using parallel analysis [36] and exploratory graph analysis [37]. We tested factor solutions ranging from one factor to the maximum number suggested by these methods. Principal axis factoring with oblique cluster rotation was used to allow for factor intercorrelations. Items with factor loadings below 0.30 were considered for removal. Internal consistencies were evaluated using McDonald’s ω, with values exceeding 0.70 considered satisfactory.

The factor structure underwent expert evaluation by the first author and two mentalization-based treatment clinicians, who prioritized achieving “simple structure” [38], given the overlapping definition of mentalizing facets. Each expert independently evaluated (1) the potential redundancy among highly correlated items, (2) the optimal number of factors, (3) the theoretical alignment between items and their respective factors, and (4) the treatment of cross-loaded and weakly-loaded items. Discrepancies were resolved through consensus discussions. Potentially problematic items were either reassigned to more appropriate factors or removed if they lacked conceptual fit, ensuring that the final solution integrated both statistical criteria and theoretical considerations.

## Results

Item analysis resulted in the removal of 11 items (S1 File, Sheets 1–2 and 1–3): four due to excessive kurtosis, five due to negative item-total correlations, and three due to redundancy. The measure’s suitability for factor analysis was confirmed by a KMO value of 0.79 and a significant Bartlett’s test of sphericity (χ²_(9316)_ = 102992, *p* < .001). Based on parallel analysis and exploratory graph analysis suggesting 10 and 8 factors, respectively, we evaluated solutions ranging from 1 to 10 factors (S1 File, Sheets 1–4–1–13).

Solutions with seven or more factors indicated over-extraction signs, as evidenced by factors with only 3–5 salient indicators, mostly uninterpretable content, and minimal additional variance explained (2–3%). Conversely, solutions with four or fewer factors showed under-extraction signs, with some factors lacking cohesive themes. The five- and six-factor solutions were comparable. Both solutions displayed strong primary loadings (Five-factor: |λ| = .24–.85, M_|λ|_ = .52; Six-factor: |λ| = .27–.91, M_|λ|_ = .53) but also contained several cross-loadings. Given the heterogeneity of the factors and their potential for further differentiation, the five-factor solution was endorsed for further partitioning.

**Fig 1:**
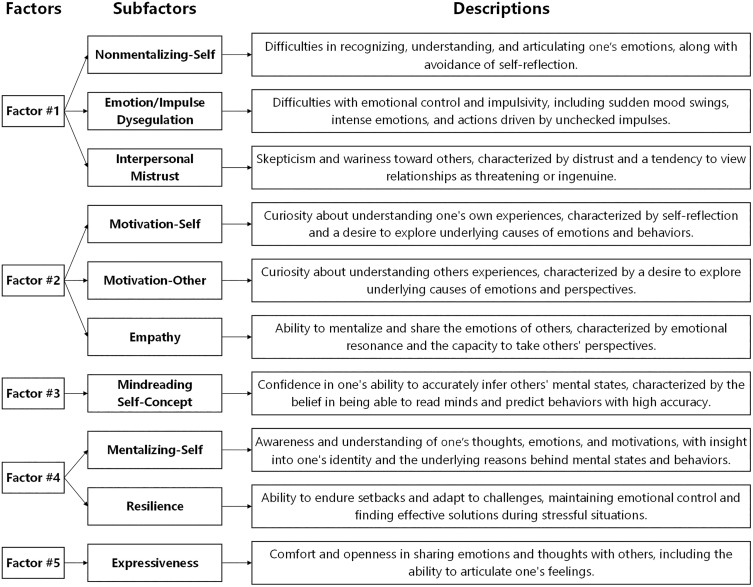
Hierarchical structure of factors, subfactors, and their descriptions.

Initial refinement of this solution involved removing one cross-loading item and reassigning seven items to more conceptually appropriate factors. Subsequently, separate EFAs were conducted on each factor, exploring one- to four-subfactor solutions. Based on both statistical and theoretical considerations, Factors #3 and #5 were retained as unidimensional, Factors #1 and #2 were each split into three subfactors, and Factor #4 was divided into two subfactors (S1 File, Sheets 1–14–1–18). The second-level EFAs led to 21 additional item removals due to weak loadings or conceptual misalignment. Moreover, several items were reassigned based on conceptual themes. The final structure comprised 115 items across 10 factors: Nonmentalizing-Self (13 items; ω = .86), Emotion/Impulse Dysregulation (15 items; ω = .85), Interpersonal Mistrust (9 items; ω = .80), Motivation-Self (9 items; ω = .84), Motivation-Others (10 items; ω = .85), Empathy (6 items; ω = .66), Mindreading Self-Concept (22 items; ω = .91), Mentalizing-Self (16 items; ω = .92), Resilience (8 items; ω = .82), and Expressiveness (7 items; ω = .83). Detailed factor descriptions are provided in Fig 1, and the complete factor structure is available in S1 File, Sheet 1–19.

Preliminary analyses were conducted to explore demographic correlates of the 10 identified factors. With regard to sex, women scored significantly higher than men on Nonmentalizing-Self (*d* = .41) and Emotion/Impulse Dysregulation (*d* = .53), and scored significantly lower on Motivation-Other (*d* = .32). For age, a significant positive correlation was found with Resilience (*r* = .16). Regarding diagnostic status, individuals with a clinical diagnosis scored significantly lower than non-diagnosed individuals on Mentalizing-Self (*p* = .03, *d* = .49). No other demographic effects were statistically significant.

## Discussion

Study 1 yielded a 10-factor structure that expands current measurement practices while aligning with theoretical dimensions of mentalizing. Mentalizing-Self/Nonmentalizing-Self represent self-oriented processes; Emotion/Impulse Dysregulation reflects affective/automatic poles; Empathy constitutes the intersections of other-oriented and affective dimensions; and Motivation factors represent elements of “mentalizing stance” [3]. Notably, Mindreading Self-Concept [terminology adopted from 12] emerged as the longest factor, suggesting existing measures may conflate genuine other-oriented mentalizing with perceived ability to “read minds”.

While several identified factors correspond with established theoretical frameworks, the emergence of Interpersonal Mistrust, Resilience, and Expressiveness as components of mentalizing warrant further consideration. These factors may represent psychological correlates of mentalizing rather than constituting its core components. Lastly, the suboptimal reliability of Empathy suggests challenges in measuring this dimension with current items. Overall, the identified 10-factor structure provides a thorough framework for measurement, enabling differentiation between adaptive and maladaptive components, distinguishing constructs along the self-other continuum, and discriminating authentic mentalizing processes from subjective assessments of one’s mentalizing capabilities.

## Study 2

### Objectives

The second phase of the study aimed to evaluate the factor structure identified in the first phase through confirmatory models, including confirmatory factor analysis (CFA), exploratory structural equation modeling (ESEM), and their bifactor counterparts. Additionally, this phase sought to examine whether the factors of the selected model could predict individuals’ dysfunction. These relationships were tested both with and without controlling for the shared variance of positive self-evaluation, following the approach used by Wendt, Zimmermann [12]. We hypothesized that the factor structure of Study 1 would be corroborated, that all factors would significantly predict dysfunction, and that these predictions would remain significant, albeit attenuated, after controlling for positive self-evaluation. The sole anticipated exception was for Mindreading Self-concept, which was expected to lose its predictive significance once positive self-evaluation was controlled for.

## Materials and methods

### Procedures, participants, and measures

The procedures for Study 2 largely paralleled those of Study 1. Participant data were collected from January 2^nd^, 2025 to January 23^rd^, 2025. A sample of 502 community-dwelling adults completed the refined 115-item set derived from Study 1 along with multiple criterion measures: the BESSI-20, FISSPD, SSFQ, and WHODAS-12 (see Table 3 for an overview of criterion measures across studies). Quality control procedures identical to Study 1 resulted in the exclusion of 51 responses. The final sample comprised 451 participants (M_age_ = 39.92, SD_age_ = 11.85, Range = 18–70; see Table 1). No missing data were present.

**Table 3:** An overview of the criterion measures.

Measure	Intended construct	# Items	Subscales	Used score
Behavioral, Emotional, and Social Skills Inventory-20 [BESSI-20; 39, 40]	Positive self-evaluation	20	Self-Management Skills, Social Engagement Skills, Cooperation Skills, Emotional Resilience Skills, Innovation Skills	Total score
Five-Item Screening Scale for Personality Disorders [FISSPD; 41]	Self functioning	5	–	Total score
Short Social Functioning Questionnaire [SSFQ; 42]	Social functioning	5	–	Total score
12-item self-administered World Health Organization disability assessment schedule 2.0 [WHODAS-12; 43]	General functioning	12	–	Total score
Screening Instrument for Borderline Personality Disorder [SI-BORD; 44]	BPD traits	5	–	Total score
Five-Factor Borderline Inventory-Screener [FFBI-Screener; 45]	BPD traits	5	–	Total score
Dark Factor of Personality-16 [D16; 46]	ASPD traits	16	–	Total score
Super Brief-Pathological Narcissism Inventory [SB-PNI; 47]	NPD traits	12	Grandiosity, Vulnerability	Grandiosity, Vulnerability
Distress Questionnaire-5 [DQ5; 48]	General distress	5	–	Total score
World Health Organization-Five Well-Being Index [WHO-5; 49]	Well-being	5	–	Total score

*Notes*. BPD = Borderline Personality Disorder, ASPD = Antisocial Personality Disorder, NPD = Narcissistic Personality Disorder.

## Analysis strategy

All analyses were conducted using Mplus [v. 8.3; 50] and BifactorIndicesCalculator package [v. 0.2.2; 51] in Rstudio. Due to the measure’s four-point ordinal response scale, we used the mean- and variance-adjusted weighted least square (WLSMV) estimator, applying oblique target rotation for ESEM models and orthogonal target rotation for bifactor-ESEM models. Given that WLSMV is robust to non-normality, no additional tests were conducted. Model selection employed a two-stage approach. First, we compared CFA and ESEM models, with ESEM selection requiring superior fit indices, lower inter-factor correlations, and well-defined factors (loadings ≥ 0.30, target loadings substantially larger than cross-loadings). Second, we compared the selected model against its bifactor counterpart, which would be preferred if it demonstrated enhanced fit, reduced cross-loadings, and a robust general factor (G-factor) with strong item loadings on either the G-factor or specific factors (S-factors) [52–54].

For bifactor models, we evaluated additional unidimensionality indices: Explained Common Variance (ECV), item-level ECV (IECV), and hierarchical omega (ω_H_). A viable G-factor required an ECV exceeding 0.70, most IECV values surpassing 0.80, and an ω_H_ above 0.80 [55–57]. Throughout the analyses, items with non-salient loadings or cross-loadings exceeding target loadings were flagged for removal. Model fit was judged using the Comparative Fit Index (CFI), Tucker-Lewis Index (TLI) (adequate ≥ 0.90), Root Mean Square Error of Approximation (RMSEA), and Standardized Root Mean Square Residual (SRMR) (adequate ≤ 0.08) [58]. Model-based reliability coefficients (ω_t_) were examined for each factor, with values above 0.70 supporting structural validity [59].

**Fig 2:**
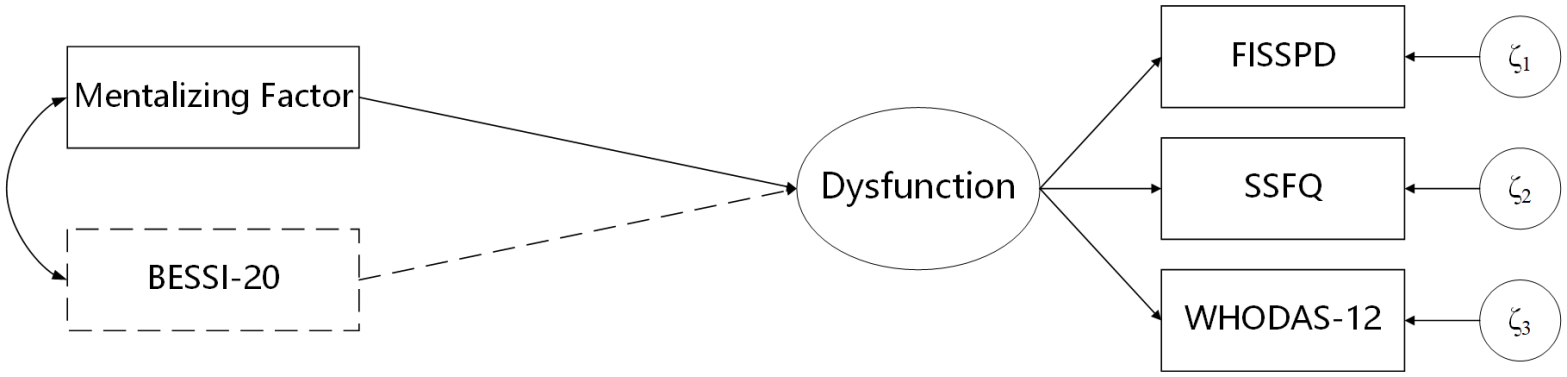
Structural models testing the identified factors’ prediction of dysfunction, modeled as a latent construct indicated by the FISSPD, SSFQ, and WHODAS-12. Two models were tested: (1) Single IV, with the factor as the sole predictor, and (2) Double IV, including the BESSI-20 as an additional predictor (dashed paths).

To examine the predictive validity of the factors, we employed SEM. Dysfunction was operationalized as a latent construct with three indicators: self dysfunction (measured by the FISSPD), social dysfunction (measured by the SSFQ), and general dysfunction (measured by the WHODAS-12), with higher scores on all indicators reflecting greater dysfunction. Two models were constructed for each factor (see Fig 2): (1) a single predictor model, where the manifest score of the factor was used to predict the latent construct of dysfunction, and (2) a double predictor model, where the manifest score of positive self-evaluation (measured by the BESSI-20) was included as an additional predictor.

## Results

The CFA model demonstrated poor fit (χ²_(6395)_ = 10363.887, *p* < .001; CFI = .887; TLI = .885; RMSEA = .037, 90% CI = [.036,.038]; SRMR = .079), while both the ESEM model (χ²_(5450)_ = 6710.105, *p* < .001; CFI = .964; TLI = .957; RMSEA = .023, 90% CI = [.021,.024]; SRMR = .036) and the bifactor-ESEM model (χ²_(5345)_ = 6491.391, *p* < .001; CFI = .967; TLI = .96; RMSEA = .022, 90% CI = [.020,.024]; SRMR = .034) exhibited good fit. The bifactor-ESEM model produced a moderately robust G-factor (|λs| = .13–.78, M_|λ|_ = .43), but ancillary indices (ECV = .37, IECV = .03–.79, ω_H_ = .85) supported the multidimensional nature of the item set.

The ESEM model demonstrated better factor differentiation with substantially lower inter-factor correlations than the CFA model (M_|r|_ = .16 vs. M_|r|_ = .40). Initial analysis revealed generally salient factor loadings (|λs| = .05–.90, M_|λ|_ = .58) and acceptable internal consistencies (ω_t_ = .77–.93), with Empathy being the exception (ω_t _= .57). Based on experts’ inspection, 13 items were deleted (10 due to weak loadings, three due to cross-loadings). The refined ESEM model showed more consistent loadings (|λs| = .23–.92, M_|λ|_ = .58), improved reliability (ω_t_ = .78 and.96; except for Empathy with ω_t_ = .57), and mostly negligible cross-loadings (|λs| = 0–.35, M_|λ|_ = .08) (S1 File, Sheets 2–11–2–13).

SEM analyses revealed that all factors except Empathy significantly predicted dysfunction, with explained variance ranging from.016 to.394 (Table 4). When controlling for positive self-evaluation, Nonmentalizing-Self, Emotion/Impulse Dysregulation, Interpersonal Mistrust, Mentalizing-Self, and Resilience maintained significant relationships (albeit with attenuated coefficients; *β* = .221–.424, *p* < .001), while Expressiveness, Motivation-Self, and Motivation-Other lost significance. Notably, all significant relationships aligned with expected directions, except for Mindreading Self-Concept, which strikingly exhibited a negative association with dysfunction.

**Table 4:** The identified factors predicting dysfunction, before and after controlling for positive self-evaluation.

	Single IV				Double IV			
Model	IV	*β* (SE)	*p*-value	R^2^	IVs	*β* (SE)	*p*-value	R^2^
1	Nonmentalizing-Self	0.551 (0.040)	<.001	0.304	Nonmentalizing-Self	0.308 (0.045)	<.001	0.576
					BESSI-20	−0.587 (0.039)	<.001	
2	Emotion/Impulse Dysregulation	0.628 (0.037)	<.001	0.394	Emotion/Impulse Dysregulation	0.415 (0.041)	<.001	0.645
					BESSI-20	−0.559 (0.038)	<.001	
3	Interpersonal Mistrust	0.573 (0.038)	<.001	0.329	Interpersonal Mistrust	0.424 (0.036)	<.001	0.667
					BESSI-20	−0.597 (0.035)	<.001	
4	Motivation-Self	−0.179 (0.061)	<.001	0.032	Motivation-Self	0.015 (0.044)	0.730	0.496
					BESSI-20	−0.699 (0.035)	<.001	
5	Motivation-Other	−0.204 (0.052)	<.001	0.041	Motivation-Other	−0.076 (0.044)	0.087	0.500
					BESSI-20	−0.731 (0.035)	<.001	
6	Empathy	−0.055 (0.051)	0.282	0.003	Empathy	−0.013 (0.031)	0.742	0.495
					BESSI-20	−0.705 (0.041)	<.001	
7	Mindreading Self-Concept	−0.128 (0.054)	0.017	0.016	Mindreading Self-Concept	0.157 (0.046)	0.001	0.516
					BESSI-20	−0.763 (0.035)	<.001	
8	Mentalizing-Self	−0.548 (0.043)	<.001	0.301	Mentalizing-Self	0.288 (0.050)	<.001	0.484
					BESSI-20	−0.475 (0.055)	<.001	
9	Resilience	−0.543 (0.038)	<.001	0.295	Resilience	0.221 (0.050)	<.001	0.529
					BESSI-20	−0.581 (0.047)	<.001	
10	Expressiveness	−0.231 (0.052)	<.001	0.053	Expressiveness	0.049 (0.042)	0.239	0.498
					BESSI-20	−0.691 (0.032)	<.001	

*Notes.* BESSI-20 = Behavioral, Emotional, and Social Skills Inventory-20. The dependent variable is dysfunction for all models. For the identified factors, factor scores were used instead of manifest scores. Fit indices and model specifications are presented in S1 File, Sheet 2–14.

## Discussion

Study 2 provided key methodological and conceptual insights about mentalizing. The superiority of ESEM over CFA demonstrated the complex, interrelated nature of mentalizing components, allowing theoretically justified cross-loadings that captured dimensional overlap and produced more realistic inter-factor correlations [53]. This modeling approach, notably absent in the original validation studies of all eight constituent measures, suggests previous CFA approaches likely overestimated factor correlations, mischaracterized relationships between components, and forced items into suboptimal factor assignments. The problematic reliability of Empathy was also replicated, which may explain its failure to predict dysfunction in single-predictor models and necessitates item addition for this factor. This low reliability suggests that the items from existing measures may not adequately capture the construct’s complexity or he limited number of items (only 6) available from existing measures to assess this complex construct. This finding necessitated our decision to develop and add new items in Study 3.

The predictive analyses revealed that different components of mentalizing relate to dysfunction in different ways when accounting for positive self-evaluation. Specifically, five components (Nonmentalizing-Self, Emotion/Impulse Dysregulation, Interpersonal Mistrust, Mentalizing-Self, and Resilience) maintained significant associations with dysfunction, suggesting these dimensions capture substantive aspects of mentalizing with genuine implications for psychological functioning. Conversely, Motivation-Self, Motivation-Other, and Expressiveness lost their predictive power after accounting for positive self-evaluation. This may reflect measurement challenges inherent in self-reporting motivational aspects of mentalizing, inevitably confounding them with self-evaluative biases. Alternatively, these motivation-related components might primarily relate to positive psychological outcomes rather than the absence of dysfunction, suggesting their importance may be more evident when examining well-being indicators rather than psychopathology markers. Future endeavors would benefit from multi-method assessments, such as observational or performance-based measures, to assess their link to actual behavior.

Perhaps the most theoretically compelling finding concerned Mindreading Self-Concept, which exhibited a negative relationship with dysfunction when controlling for positive self-evaluation. This finding substantiates a core assumption of mentalizing theory: the adaptive value of humility in understanding others’ minds. The mentalizing stance, characterized by curiosity, openness, and tolerance for uncertainty about mental states, directly contrasts with an overly confident sense that one comprehends others’ minds with certainty [3]. The negative association between Mindreading Self-Concept and dysfunction suggests that, once positive self-evaluation is accounted for, excessive confidence in one’s mindreading abilities may actually reflect a non-mentalizing mode characterized by rigid certainty rather than the flexible curiosity that facilitates genuine understanding of others.

## Study 3

### Objectives

Our third study had three primary objectives: (1) to cross-validate the factor structure established in Study 2 using an independent sample, (2) to add items to Empathy, examine their performance, and evaluate the reliability of this revised factor, and (3) to assess the criterion-related validity of the identified factors through their associations with traits of borderline personality disorder (BPD), narcissistic personality disorder (NPD), and antisocial personality disorder (ASPD), as well as general distress and well-being. Empathy was singled out for refinement because it was the only factor to demonstrate unacceptable internal consistency (ω_t_ = .57) in Study 2.

## Materials and methods

### Procedures, participants, and measures

Study 3 maintained methodological continuity with the previous studies while expanding the measurement battery. Participant data were collected from February 7^th^, 2025 to March 2^nd^, 2025. Participants completed the revised item pool from Study 2 alongside the BESSI-20, SI-BORD, FFBI-SSF, D16, SB-PNI, DQ5, and WHO-5 (see Table 3). Regarding the addition of items to Empathy, the research team developed and evaluated new items through a systematic process. The first author initially generated 20 candidate items, which underwent expert and co-author review. Five items were ultimately selected based on two primary criteria: (1) comprehensive coverage of empathy facets and (2) discriminant validity from the related factors (Motivation-Other and Mindreading Self-Concept).

We initially recruited 342 community-dwelling adults. Following quality control procedures identical to Study 1, 36 responses were excluded, yielding a final sample of 306 participants (M_age_ = 34.22, SD_age_ = 13.37, Range = 18–70; see Table 1). No missing data were present.

## Analysis strategy

All analyses and visualizations were performed using Mplus [v. 8.3; 50], IBM SPSS [v. 27; 31], and packages ggplot [v. 3.5.1; [60] and corrplot [v. 0.95; [61] in Rstudio. The item analysis procedure for the new items, as well as the fit of the ESEM model, was examined using the same procedure as in previous studies. Item retention decisions were based on parameter estimates using criteria consistent with Study 2. However, based on the judgment of experts, exceptions could be made for items with strong loadings (λ ≥ .50) in Study 2 but weaker loadings in the current dataset. Additionally, items displaying persistent cross-loadings or weak loadings across both studies were omitted.

Criterion-related validity was assessed by calculating Pearson correlation coefficients between the identified factors and the relevant constructs. Additionally, partial correlations were computed, controlling for positive self-evaluation (measured by the BESSI-20). For the identified factors, factor scores were used instead of manifest scores to optimize measurement precision [54]. The magnitudes of the Pearson coefficients were interpreted as small, medium, and large for *r* = .10, *r* = .20, and *r* = .30, respectively [62,63]. To assess the stability of these correlations, we computed bootstrapped confidence intervals using the bias-corrected accelerated method with 1000 resamples.

## Results

All new items performed well in item analysis, except one (*If I realize I have hurt or upset a loved one, I would feel guilty*) that demonstrated elevated kurtosis of 4.01 (S1 File, Sheet 3−1). Despite this statistical deviation, the item was retained based on expert recommendation, given its essential role in assessing adaptive guilt.

The initial ESEM model demonstrated good fit (χ^2^_(4646)_ = 5422.114, *p* < .001; CFI = 0.957; TLI = 0.947; RMSEA = .023, 90% CI = [.020,.026]; SRMR = .042) and relatively well-defined factors (|λs| = .05–.89, M_|λ|_ = .54; S1 File, Sheets 3–2–3–4). However, subsequent examinations helped identify several items requiring removal due to either weak loadings or problematic cross-loadings. The refined ESEM model also exhibited good fit (χ^2^_(3560)_ = 4222.868, *p* < .001; CFI = 0.959; TLI = 0.949; RMSEA = .025, 90% CI = [.022,.028]; SRMR = .041), salient factor loadings (|λs| = .19–.88, M_|λ|_ = .58), desirable factor differentiation (M_|*r*|_ = .17, |*r*| = .01–.47), and acceptable reliability (ω_t_ = .72–.94) (S1 File, Sheets 3–5–3–7). Notably, including the five new items enhanced the reliability of Empathy substantially (ω_t_ = .81; see Table 5).

**Table 5:** Four highest-loading items and their standardized factor loadings for the identified factors (final structure).

Item	Item/description	λ	Item	Item/description	λ
**Nonmentalizing-Self (9 items; ω**_**t**_ ** = .85)**					
MentS22	Challenges in verbal articulation of emotional experiences	0.630	MZQ11	Limited capacity to fully experience the depth or strength of one’s emotional responses	0.615
MentS21	Imprecise differentiation between various emotional states experienced	0.626	MentS18	Struggles to acknowledge negative feelings to oneself	0.614
**Emotion/Impulse Dysregulation (10 items; ω**_**t**_ ** = .89)**					
RFQ4	When I get angry I say things that I later regret	0.833	RFQ3	When I get angry I say things without really knowing why I am saying them	0.794
MMQ2	I am an impulsive person	0.805	MMQ7	I sometimes feel like I am losing control of my emotions	0.775
**Interpersonal Mistrust (6 items; ω**_**t**_ ** = .72)**					
MMQ29	It’s better to beware of strangers	0.803	MMQ20	I don’t trust others	0.352
MMQ13	It’s better to beware of others	0.762	MMQ27	People abandon me	0.30
**Motivation-Self (6 items; ω**_**t**_ ** = .77)**					
MMQ18	I often think about why things happen	0.731	MMQ17	I find beneficial to analyse my behaviour	0.600
MMQ16	I ponder over what happens to me	0.620	MMQ32	I’m keen on understanding why certain things happen to me	0.427
**Motivation-Other (7 items; ω**_**t**_ ** = .84)**					
MEQ14	I find it helpful to think about the reasons for others’ emotions	0.780	MEQ12	I think it is enriching to recognize emotions in others	0.586
MEQ16	I find it exciting to think about where others’ emotions come from	0.683	MEQ10	I am interested in the emotions of others	0.580
**Empathy (9 items; ω**_**t**_ ** = .81)**					
MMQ14	I’m able to empathize with others when they tell me something	0.734	NEW3	If I see my friend is not doing well, I try to be by their side and make them feel safe.	0.561
MentS6	Capacity to empathize with others’ emotions	0.636	NEW5	I would feel guilty if I realize I have hurt or upset a loved one.	0.541
**Mindreading Self-Concept (19 items; ω**_**t**_ ** = .94)**					
MMQ5	I can tune in other people’s mental states	0.854	MMQ4	I’m able to get the deepest aspects of people around me	0.766
CAMSQ19	Interpreting emotions from others’ facial expressions	0.779	CAMSQ10	Recognizing when others conceal their thoughts	0.746
**Mentalizing-Self (16 items; ω**_**t**_ ** = .91)**					
IMQ24	I have high confidence in knowing who I am	0.721	CAMSQ9	Comprehension of one’s emotions	0.668
CAMSQ14	Awareness of reasons behind bad moods	0.712	CAMSQ7	Understanding reasons for personal interests	0.636
**Resilience (6 items; ω**_**t**_ ** = .86)**					
MMQ30	I am able to cope with difficult situations	0.778	MMQ24	I am able to sort out difficult problems when life presents those to me	0.728
MMQ25	I am able to bear the emotional load of stressful situations	0.740	MMQ26	When I feel an intense emotion, I can control it	0.665
**Expressiveness (7 items; ω**_**t**_ ** = .90)**					
MEQ7	I can explain my different emotions to others	0.883	MEQ6	I find it exciting to talk about my emotions with others	0.785
MEQ8	I think it is useful to talk about my emotions	0.803	MentS16	Frequent discussions about feelings with close individuals	0.677

*Notes.* CAMSQ = Certainty About Mental States Questionnaire, FIMI = Four-Item Mentalising Index, IMQ = Interactive Mentalizing Questionnaire, MentS = Mentalization Scale, MEQ = Mentalizing Emotions Questionnaire, MMQ = Multidimensional Mentalizing Questionnaire, MZQ = Mentalization Questionnaire, RFQ = Reflective Functioning Questionnaire. To address copyright considerations, instead of the full items of the MZQ, MentS, FIMI, and CAMSQ, item descriptions are provided (see Supplements, Contents sheet).

**Fig 3:**
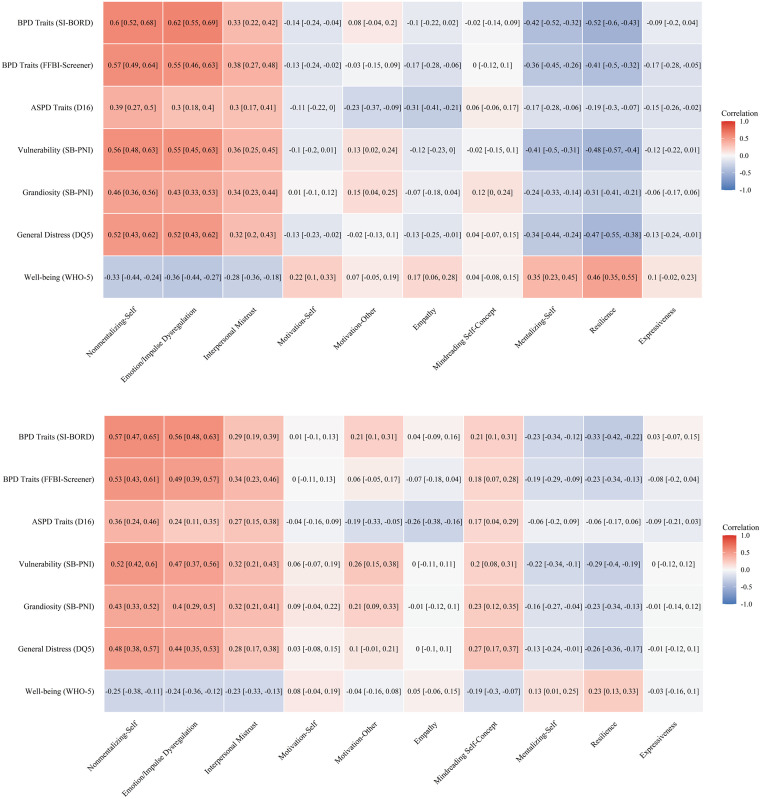
Zero-order correlations (upper panel) and partial correlations controlling for positive self-evaluation (lower panel) between the identified factors and criterion variables.

Criterion-related validity analyses mostly indicated strong associations between the identified factors and the criterion constructs (Fig 3). Notably, Nonmentalizing-Self, Emotion/Impulse Dysregulation, Interpersonal Mistrust, Mentalizing-Self, and Resilience showed large correlations with personality disorder traits, general distress, and well-being. Empathy and Motivation-Other were particularly linked to ASPD traits, with large and medium magnitudes, respectively. These associations were consistent after controlling for positive self-evaluation, yet the strength of most correlations diminished under this adjustment. Lastly, Motivation-Self and Expressiveness exhibited weak to moderate correlations with the criterion variables, highlighting their relatively small contributions.

Two important suppression effects were observed: For Mindreading Self-Concept, negligible bivariate correlations with criterion measures transformed into moderate associations (positive with pathology, negative with well-being) after controlling for positive self-evaluation. Similarly, Motivation-Other’s modest correlations with NPD traits strengthened substantially when accounting for positive self-evaluation. To ensure the stability of the regression estimates, the correlation between the two observed predictors in each double-predictor model was examined. The correlations were found to be in the *r* = .10 –.64 range (M_|r|_ = .36; lowest for Empathy and highest for Mentalizing-Self), indicating that multicollinearity was not a confounding factor in the analysis.

## Discussion

Study 3 successfully cross-validated the proposed 10-factor structure, though several items required removal due to weak loadings or cross-loadings, likely reflecting the sample-specific variability and/or the overlap between mentalizing dimensions. Criterion-related validity analyses revealed that Nonmentalizing-Self, Emotion/Impulse Dysregulation, Mentalizing-Self, Resilience, and Interpersonal Mistrust demonstrated robust associations with personality disorder traits, general distress, and well-being—which were largely maintained after controlling for positive self-evaluation. These findings support the relationship between mentalizing and personality dysfunction as described in the Alternative Model of Personality Disorders [64]. Self functioning relies on self-oriented mentalizing processes that enable coherent self-representations, emotion regulation, and goal-directed behavior. Effective Mentalizing thus forms the foundation for integrated identity and self-other distinction, both essential components of adaptive functioning. Interpersonal functioning similarly depends on other-oriented mentalizing that allows individuals to understand others’ experiences, appreciate different perspectives, and recognize the impact of their actions. This other-oriented mentalizing facilitates the development of mutually rewarding relationships characterized by empathic understanding and emotional intimacy [65,66].

The differential associations of the motivation dimensions and suppression effects highlight the complex nature of mentalizing. Motivation-Self showed moderate associations with well-being but remained relatively independent from psychopathology markers, suggesting that curiosity about one’s mental states may primarily operate as a resilience factor within the domain of psychological wellness rather than psychopathology. Empathy demonstrated strong negative correlations with ASPD traits, both before and after controlling for positive self-evaluation, aligning with the conceptualization of ASPD as fundamentally characterized by callousness and disregard for others [64]. Similarly, Motivation-Other demonstrated a specific negative correlation with ASPD traits but negligible relationships with other variables, suggesting a particular association between disinterest in others’ experiences and antisocial tendencies [3]. Regarding suppression effects, partial correlations for Mindreading Self-Concept revealed direct associations with personality disorder traits and general distress, and inverse associations with well-being. Congruent with the findings of Study 2, this pattern only became apparent when controlling for positive self-evaluation, suggesting the counterproductive effects of overconfidence in one’s mindreading ability. On the other hand, the strengthened association between Motivation-Other and narcissistic traits after controlling for positive self-evaluation suggests that narcissistic individuals’ interest in others’ minds may represent strategic information-gathering rather than genuine interpersonal curiosity, consistent with abovementioned findings on ASPD traits.

## General discussion

Our three sequential studies investigated the common structure of mentalizing by analyzing a combined pool of items, yielding a validated 10-factor structure that captures its multidimensional nature. The measure’s factor structure clarified conceptual boundaries between core mentalizing processes and related psychological constructs. The combination of pre-existing measures not only provided more comprehensive assessment but also clarified conceptual boundaries between core mentalizing processes and related psychological constructs. Notably, Interpersonal Mistrust and Resilience factors were solely built upon the items of the MMQ [22], perhaps reflecting the conceptual biases of their source measures rather than the central construct itself. This strengthens the argument that they represent correlates rather than fundamental components of mentalizing. The superiority of ESEM over CFA models further demonstrates the inherently overlapping nature of mentalizing dimensions, revealing how previous endeavors may have mischaracterized interrelated aspects or may have misplaced items [54]. Perhaps most significantly, our findings highlight how self-report assessment of mentalizing is differentially affected by self-evaluation biases, with positively worded dimensions showing greater contamination, while core factors like Nonmentalizing-Self and Emotion/Impulse Dysregulation remain relatively unaffected (Fig 3). This methodological insight helps reconcile contradictory findings in previous literature and suggests a critical refinement for future research: controlling for positive self-evaluation may be necessary for accurate assessment of adaptive mentalizing dimensions.

One of our most theoretically compelling findings concerns Mindreading Self-Concept emerging as a long, distinct factor that, after controlling for positive self-evaluation, surprisingly shows direct associations with dysfunction and psychopathology markers. This paradox aligns with the findings of Wendt, Zimmermann [12]: higher self-reported mindreading uniquely predicted poorer psychosocial functioning when controlling for positive self-evaluation, suggesting this is not just a measurement artifact but reflects a fundamental aspect of mentalizing processes. Similar patterns emerged in Heine, Schmukle [67], where individuals with strong self-insight motives actively sought self-knowledge yet showed no better self-perception accuracy than those with weaker motives. Together, these findings point to a counterintuitive conclusion: confidence in reading others’ minds often reflects not superior mentalizing but rather its absence.

This may indicate a form of hypermentalizing, which involves drawing overly elaborate and often incorrect conclusions about the thoughts and intentions of others [3]. Indeed, Bilotta, Carcione [68] found that individuals with narcissistic personality disorder believe they are highly skilled at assessing their own and others’ mental states, even though they show significant deficits in this area. These empirical observations align with multiple theoretical perspectives. From a psychodynamic perspective, Kernberg [69] describes how such overconfidence serves as a defense, where a pathological grandiose self (built on idealization, devaluation, and projection) protects an individual from underlying feelings of shame and fragility. From a cognitive perspective, von Hippel and Trivers [70] propose that self-deception evolved primarily as a strategy for interpersonal persuasion. Supporting this, Mei, Ke [71] demonstrate that distorted metacognitive processes allow individuals to maintain inflated beliefs without conscious awareness of contradictory evidence. This mirrors the Dunning-Kruger effect, where studies show that the most unskilled individuals are the least aware of their incompetence, including in the domain of emotional intelligence [72,73]. Thus, once self‐esteem is held constant, high Mindreading Self‐Concept appears to index self‐deceptive narcissistic patterns rather than actual skill, highlighting that true mentalizing thrives on the humility of a ‘not‐knowing’ stance [3]. While genuine skill may contribute to high Mindreading Self-Concept scores in some cases, the consistent association with dysfunction markers suggests pathological overconfidence as the predominant driver.

The differentiated associations between identified factors and personality pathology provide empirical validation for theoretical formulations about mentalizing’s role in personality functioning. Nonmentalizing-Self, Emotion/Impulse Dysregulation, and Mentalizing-Self demonstrated the strongest associations with all personality disorder measures, confirming their centrality to personality functioning as emphasized in dimensional models [74]. Moreover, The differential association patterns of Empathy and Motivation dimensions with criterion variables suggest they may serve specialized adaptive functions rather than simply being weaker predictors of dysfunction: lack of Empathy appearing as a specific risk factor for ASPD traits [75], Motivation-Self functioning primarily in the domain of psychological growth and well-being rather than psychopathology prevention, and Motivation-Other revealing a complex dual nature where it may reflect either genuine interpersonal curiosity or strategic information gathering depending on personality structure [3].

Practically, this multidimensional framework enables more sophisticated assessment of mentalizing profiles, facilitating more precise case formulation and tailored interventions across the treatment process. Clinicians could use patients’ profiles to identify specific intervention targets. For instance, high scores on Emotion/Impulse Dysregulation might indicate a need for emotion regulation skills training, while elevated Interpersonal Mistrust would suggest focusing on attachment-based interventions to build epistemic trust and safety. The framework also allows tracking therapeutic progress by monitoring changes in specific factor scores over time, providing more fine-grained outcome assessment than global mentalizing measures. For personality disorders specifically, assessing these factors may provide nuanced discrimination between superficially similar presentations (e.g., distinguishing narcissistic individuals with high Mindreading Self-Concept but poor actual mentalizing from those with genuine mentalizing) while also identifying transdiagnostic vulnerabilities like self-reflection deficits and emotion dysregulation that cross diagnostic boundaries [76].

Several limitations should be acknowledged. First, the sample size in Study 1 was modest for EFA analyses, although the resulting factor structure was subsequently cross-validated in the larger samples of Studies 2 and 3. Second, our reliance on community samples rather than clinical populations limits conclusions about the clinical utility of this factor structure in detecting psychopathology or measuring therapeutic change. Third, the cross-sectional design precludes causal inferences regarding the relationships between mentalizing dimensions and dysfunction/pathology. Fourth, the cultural specificity of our Iranian samples potentially affects the generalizability of findings to other cultural contexts. Fifth, we were unable to assess test-retest reliability due to our cross-sectional design. Future research should examine temporal stability of all factors, with special attention to Empathy.

Sixth, our analytical approach treated positive self-evaluation solely as a statistical confound following Wendt, Zimmermann [12] framework. However, we acknowledge that this decision reflects one theoretical perspective among several possibilities. Positive self-evaluation could alternatively be conceptualized as an integral component of self-related mentalizing, where maintaining a balanced yet positive self-view might itself reflect adaptive mentalizing capacity. Future research should explore whether positive self-evaluation functions as measurement bias, a protective factor moderating the impact of mentalizing deficits, or a core component of the mentalizing construct itself. Such investigations could employ mediation analyses, moderation models, or expanded factor structures that incorporate self-evaluative processes as an additional mentalizing dimension rather than a confound. Additionally, since the final item set is largely compiled from existing instruments, it inevitably inherits some of their common shortcomings (e.g., essential aspects such as body-related mentalizing are missing). Future research should also include measurement invariance testing across sociodemographic groups to allow valid group comparisons. Finally, given the considerable length of the item pool used to derive this structure, identifying the most salient items/concepts to develop a brief assessment tool based on this framework is a necessary next step.

## Supporting information

S1 File Detailed results of analyses for all three studies. (XLSX)
